# Neighbourhood Poverty, Work Commitment and Unemployment in Early Adulthood: A Longitudinal Study into the Moderating Effect of Personality

**DOI:** 10.1371/journal.pone.0167830

**Published:** 2016-12-09

**Authors:** Jaap Nieuwenhuis, Rongqin Yu, Susan Branje, Wim Meeus, Pieter Hooimeijer

**Affiliations:** 1 OTB–Research for the Built Environment, Faculty of Architecture, Delft University of Technology, Delft, the Netherlands; 2 Department of Psychiatry, University of Oxford, Oxford, United Kingdom; 3 Research Centre Adolescent Development, Faculty of Social and Behavioural Sciences, Utrecht University, Utrecht, the Netherlands; 4 Urban and Regional research centre Utrecht (URU), Faculty of Geosciences, Utrecht University, Utrecht, the Netherlands; University of Granada, SPAIN

## Abstract

We studied how personality moderates the effect of neighbourhood disadvantage on work commitment and unemployment in early adulthood. Using a personality typology of resilients, overcontrollers, and undercontrollers, we hypothesised that the association between neighbourhood poverty and both work commitment and unemployment would be stronger for overcontrollers and undercontrollers than for resilients. We used longitudinal data (N = 249) to test whether the length of exposure to neighbourhood poverty between age 16 and 21 predicts work commitment and unemployment at age 25. In line with our hypothesis, the findings showed that longer exposure was related to weaker work commitment among undercontrollers and overcontrollers and to higher unemployment among undercontrollers. Resilients’ work commitment and unemployment were not predicted by neighbourhood poverty.

## Introduction

Growing up in neighbourhoods with high levels of poverty is often thought to have a negative impact on occupational outcomes later in life (e.g., [1–5]). With regard to occupational outcomes, these neighbourhood effects have mainly been studied by examining the relation between neighbourhood disadvantage and unemployment [6,7]. The relation between neighbourhood disadvantage and unemployment may be understood through the socialisation mechanism: because youth in poor neighbourhoods are more likely to be exposed to unemployment in their local environment than youth in more affluent neighbourhoods, youth in poor neighbourhoods may adopt lower work commitment, which may lead them to become unemployed as well. From this logic it follows that attitudes towards work play an important role. However, so far, neighbourhood effects studies of occupational outcomes have mainly looked at unemployment. In order to extend our understanding of this process, we will examine the relation between neighbourhood adversity and both unemployment and work commitment. This will allow us to not only to look at ‘hard’ measures of occupational outcomes, but also to examine how environmental disadvantage might be related to attitudes.

### Neighbourhood poverty and occupational outcomes

Exposure to neighbourhood poverty is generally considered to predict negative occupational outcomes for youth (e.g., [1–5]). In the literature, various ideas exist about the possible mechanisms behind this neighbourhood effect [8]. One prominent developmental principle is the socialisation mechanism, which suggests that social behaviour is learned through conditioning and imitation of other’s behaviour [9]. Applying this theory to the neighbourhood context–we would expect that adolescents are likely to incorporate norms that are dominant amongst neighbourhood residents [10,11]. Poor neighbourhoods are likely to contain more adult residents who are unemployed or not in the labour force compared to more affluent neighbourhoods [12]. This suggest that poor neighbourhoods are less likely to contain positive role models who are able to demonstrate the benefits of employment and are instead more likely to contain residents with lower job satisfaction [13]. Furthermore, during adolescence, youths start to spend less time with parents and more with peers [14]. In poor neighbourhoods, this increased contact with neighbourhood peers increases the likelihood that adolescents come in contact with negative role models in the neighbourhood. With prolonged exposure to neighbourhood poverty during their formative years, youth may internalise negative attitudes towards work. These internalised negative attitudes could result in a higher odds to become unemployed in early adulthood.

Another factor that could explain the relation between neighbourhood poverty and occupational outcomes is the social capital that people can access through their social network. It is argued that in poor neighbourhoods, residents often have more homogeneous contacts, while residents of affluent neighbourhoods often have more heterogeneous contacts [15]. More heterogeneous social contacts often can provide more varied information, while in a more homogeneous social network, social contacts are more likely to know the same people, and therefore have access to the same information. For instance, in poor neighbourhoods, where people have more homogeneous social networks, residents are less likely to have access to new information about jobs and opportunities. As a result, adolescents are less likely to build up social networks that are able to provide new job information or can help them find a job [16], therewith increasing their chances for unemployment in early adulthood.

The above theories suggest that exposure to neighbourhood poverty could result in weak work commitment for residents of the neighbourhood. Previous studies have already looked at ‘hard’ occupational outcomes: links have been found between neighbourhood poverty and income [17,18] and unemployment [6,7]. However, how neighbourhood poverty is related to residents’ attitudes has not been studied before. The concept of work commitment has been associated with unemployment [19], school commitment, and educational level [20]. By examining work commitment in a neighbourhood context, we aimed to gain more insight into how neighbourhood poverty is related to unemployment, as well as to an attitudinal occupational measure (i.e., work commitment), that is associated with unemployment.

### Moderating role of personality

Although exposure to neighbourhood poverty is in general linked to negative occupational outcomes, the associations may be different for individuals with different personalities; that is, not all youths’ occupational outcomes will be equally affected by their neighbourhood. Some youth may have negative occupational outcomes because they are more vulnerable to the negative influences of the neighbourhood they lived in, while other youth may still have good occupational outcomes despite growing up in a neighbourhood characterised by poverty (e.g., [21]). Because youth with different personalities may differ in their responses to poor neighbourhoods, these youths may also differ in their occupational outcomes.

An influential typology of personality distinguishes three types: resilients, overcontrollers, and undercontrollers [22]. These three personality types differ in the levels of ego-control and ego-resiliency. Ego-control refers to the tendency to contain versus express motivational impulses. Ego-resiliency refers to the tendency to respond flexibly versus rigidly to environmental demands. Whereas resilients respond relatively more adaptively and flexibly, both overcontrollers and undercontrollers are relatively inflexible in reacting to environmental challenges [22]. Hence, resilients may be less susceptible to the influence of the neighbourhood than the less flexible overcontrollers and undercontrollers.

This personality typology is ideal for studying individuals’ reactions to environmental challenges such as negative neighbourhood environments [23,24]. We expect that neighbourhood adversity will interact with individuals’ personality, provoking individuals with non-resilient personality types (i.e., overcontrollers and undercontrollers) to have more negative occupational outcomes when they grow up in poor neighbourhoods when compared with resilient individuals.

To summarise, we first hypothesise that exposure to neighbourhood poverty will be associated with weaker work commitments and higher odds of unemployment. Second, we expect that in poor neighbourhoods, individuals with non-resilient personality types will be more at risk to have weaker work commitments or to become unemployed than individuals with a resilient personality type. Therefore, we hypothesise that the association between exposure to neighbourhood poverty and work commitments and unemployment will be weaker for resilients than for overcontrollers and undercontrollers.

## Method

### Participants

Participants were 249 Dutch youths from the middle-to-late adolescent cohort of the CONAMORE sample who were not in full-time education during early adulthood and had a job (i.e., at risk of becoming unemployed; n = 232) or were unemployed (n = 17). They were part of an ongoing panel study CONflict And Management Of RElationships study (CONAMORE). For participation in the present study, written informed consent was obtained from adolescents and their parents, and also from all the participating schools. Treatment of participants was in accordance with the ethical standards of the APA. The middle-to-late adolescent cohort of the CONAMORE sample consisted of 390 respondents recruited from various high schools in the province of Utrecht, the Netherlands. In total, 20 randomly selected schools in Utrecht were selected, of which 12 decided to participate. From these schools, more than 99% of the approached students decided to participate. The sample had an average age of 16.7 (range 16–20) years at the first wave. 40% were males, 60% were females. There was an underrepresentation of ethnic minorities: 12.6% in our sample vs. 22% in the Netherlands [25]. In waves 1, 2, 3, 4, 5 and 6 the number of respondents was 390, 390, 370, 369, 362, and 291, respectively. The first five waves of the CONAMORE were collected annually, starting in 2001. The sixth wave was collected in 2010 and included an additional Life History Calendar (LHC; [26]) with retrospective questions from the age of 12 until the sixth wave (average age 25). The LHC contained questions about where respondents lived, when they finished education, and whether they have been (un)employed. For the first five waves, sample attrition was very low (7% from wave 1 to 5). Attrition for the sixth wave was higher (20%), because of the 5-year time gap between wave five and six, compared to the one-year gap between the earlier waves. We used data from all waves. We focused on testing the relation between poverty in the adolescent neighbourhood and employment outcomes for young adults who were not in full-time education anymore, because only after entering the labour market there can be a risk for unemployment. As most adolescents go to school until about age 21 (especially in higher vocational and scientific education), wave 5 included a large sample of respondents who were still in school; at wave 6 (average age 25), most respondents finished education (N = 249). Because the question for work commitment was not asked to respondents that were unemployed at the time of wave 6, the sample size for work commitment was slightly smaller (N = 232).

### Measurements

Work commitment. Work commitment was measured at the sixth wave for respondents who had a job (N = 232), using the Utrecht-Management of Identity Commitments Scale (U-MICS; [27]), which consisted of five items to measure the degree to which adolescents derive self-confidence from the occupational choices they made, with response categories 1 (completely true) to 5 (completely untrue). The items are (translated from Dutch): “My work makes me feel confident about myself”; “My work gives me certainty in life”; “Because of my work I feel certain about myself”; “My work gives certainty for the future”; and “Because of my work I can perceive the future optimistically”. We reverse coded the answers and constructed a scale for work commitment with high reliability (Cronbach’s *α* = .92).

Unemployment. For unemployment, we used the LHC to construct a dichotomous variable measuring whether respondents, after leaving full-time education, have been consecutively unemployed for three months or more (1, n = 27) or not (0, n = 222), at the time of the sixth wave.

The LHC data was geo-coded, and included all six-digit postcodes where respondents lived between the age of 12 and the time of the sixth wave (average age 25). Six-digit postcode areas contain, on average, 17 households, and range from 0–1485 households. In our sample, respondents did not cluster in postcodes, with on average 1.04 respondents per postcode area when the respondents were 16 years old. This enabled us to merge the individual-level data with neighbourhood characteristics on the postcode-level as provided by Statistics Netherlands [28].

Exposure to neighbourhood poverty. To measure exposure to neighbourhood poverty, we used the average property value measured in 2004. The average property value of dwellings in the neighbourhood was used as a proxy to measure neighbourhood wealth, because it captures the quality of the dwelling and the social and physical attributes of the neighbourhood [29]. The variable was measured at the scale of six-digit postcode areas, which is a good scale to measure socialisation, because socialisation is more likely to happen through neighbours in close proximity than through neighbours living blocks away [30]. To measure exposure, we calculated the number of months respondents lived in neighbourhoods in the lowest quintile of wealth (i.e., the poorest neighbourhoods), between the ages of 16 and 21. We chose these years because the parental neighbourhood may be more informative than the neighbourhood where people lived during early adulthood, because the latter can likely be seen as a transitional neighbourhood during the period of higher education. The continuous variable ranged from 0 to 1, where 0 indicated no exposure, 1 indicated that the respondent was exposed to neighbourhood poverty during the entire period from age 16 to 21, and values between 0 and 1 indicated exposure to neighbourhood poverty as the proportion of the period from age 16 to 21.

Personality. Personality was assessed annually for five years with the Quick Big Five questionnaire [31,32]. Thirty personality markers were used to assess five personality dimensions (each with 6 items): extraversion (e.g., “talkative”), agreeableness (e.g., “sympathetic”), conscientiousness (e.g., “systematic”), emotional stability (e.g., “worried”, reverse-scored), and openness to experience (e.g., “creative”). Adolescents rated their personality on a 7-point Likert scale ranging from 1 (very untrue) to 7 (very true). Various studies have reported adequate reliability and validity of this scale (e.g., [33]). In the current study, across wave 1 to wave 5, Cronbach’s *α* ranged from .80 to .87 for extraversion, from .81 to .87 for agreeableness, from .85 to .91 for conscientiousness, from .80 to .83 for emotional stability, and from .76 to .77 for openness to experience. Several studies have shown that Block and Block’s [22] three personality types (i.e., overcontrollers, undercontrollers, and resilients) can be constructed directly from the Big Five dimensions [34–36]. An earlier study constructed types of personality development with Latent Class Growth Analysis (LCGA; [37]) on the original 1313 cases, including the current sample [38]. This method calculates the most common configurations of the Big Five personality dimensions within individuals, taking into account their developmental trajectory over the five waves of data collection. In the current research, we adopted Branje et al.’s [38] classification of personality types. The Big Five profiles of these three personality types were consistent with those of other studies (e.g., [39,40]). See Branje et al. [38] for specific scores on Big Five traits for each personality type. In our sample, there were 83 (33.3%) overcontrollers, 70 (28.1%) undercontrollers, and 96 (38.6%) resilients. This was quite similar compared to the overall sample where the percentages were: overcontrollers: 33.5%, undercontrollers: 30.0%, resilients: 36.5%.

Control variables. We used sex and highest achieved educational qualification as individual-level control variables. Sex was a dummy variable (male = 0 (40%); female = 1 (60%)). Education was measured as an ordinal variable with categories, from lowest to highest: 1) high school or lower (20%), 2) middle-level vocational education (20%), 3) higher vocational education (29%), and 4) university (31%).

In order to control for the family background we included parental education, parental unemployment, and parental ethnicity. Parental education was measured as a set of six dummy variables, including: 1) lower vocational education or lower (5%); 2) preparatory middle-level vocational education (8%); 3) middle-level vocational education (19%); 4) higher general continued education or preparatory scientific education (10%); 5) higher vocational education (23%); and 6) university or higher (35%). Parental unemployment was measured as a dummy where 1 indicated that the family’s breadwinner had been unemployed during the period where the respondent was between 16 and 18 years old (12%), and 0 not unemployed (88%). Parental ethnicity was also measured as a dummy (1 = both parents were foreign born (11%); 0 = else (89%)).

### Analytical method

In our analyses, the outcome variables unemployment and work commitment were both measured at the sixth wave, when respondents were on average 25 years of age. Both personality and exposure to neighbourhood poverty were measured over the period of the first five waves, i.e., between the ages 16 and 21. In this way, exposure to neighbourhood poverty represents a lag that allows us to test the effect of exposure in middle-to-late adolescence on outcomes in early adulthood. We conducted the analyses in Stata 13 [41].

The variables work commitment and education had missing cases. To test whether these cases were missing completely at random (MCAR) we performed Little’s MCAR test [42]. This test did not reject the MCAR assumption (*χ*^2^(2) = 5.04, *p* = .08), meaning we could impute the missing cases. We used Stata’s “mi impute chained” command that imputes multiple variables by using chained equations, and takes into account the auxiliary variables (i.e., exposure to neighbourhood poverty, personality, sex, parental education, parental unemployment, and parental ethnicity; [43,44]). We used 20 imputations. Work commitment had 224 non-missing cases and 8 were imputed (3%). Education had 213 non-missing cases and 19 were imputed (8%). The imputed sample had a larger proportion of resilients, males, respondents with highly educated parents, and respondents with foreign parents. We included all variables in the imputation process as auxiliary variables in order to account for the differences between the imputed and the non-imputed sample. The presented results are pooled results using Rubin’s combination rules [45], and were obtained using Stata’s “mi estimate” command.

Because of the measurement levels for the dependent variables, we used two models: for the dichotomous variable unemployment we used logistic regression, and we used linear regression for work commitment. Although there was no clustering in our data, some respondents lived in the same postcode area (on average 1.04 respondents per postcode). To deal with this, we reported the results of the analyses in which we clustered the standard errors on the six-digit postcode area in which respondents lived when they were 16 years old. The effect size, direction, and significance levels of the results from the analyses with and without clustering were consistent. To test whether adolescents with different personalities experienced different neighbourhood effects, we employed interaction effects between personality and exposure to neighbourhood poverty.

It should be noted that neighbourhoods are not random selections of households. Instead, households sort into neighbourhoods based on the preferences and constraints in the household, such as preferences for neighbourhoods close to certain amenities and financial constraints. When this selection bias is neglected, neighbourhood effects could be misspecified [46,47]. Since we study neighbourhoods during adolescence, selection bias in our analyses may be minimal, as children usually do not select their own neighbourhoods, but their parents decide when and where to move. However, it is possible that a selection bias is present through the parents. We tried to take parental selection into account in our models by including a whole range of family characteristics, which might explain neighbourhood choice, such as parental education, parental unemployment, and parental ethnicity.

## Results

We examined whether adolescents with different personality types had different scores on the three key variables: exposure to neighbourhood poverty, work commitment, and unemployment. Table 1 shows the descriptive statistics for each personality type, which revealed minor differences on the three key variables. We conducted a series of tests to examine differences between the personality types, but found no significant differences between the personality types in neighbourhood poverty (ANOVA: *F*(2) = .50, *p* = .61), work commitment (ANOVA: *F*(2) = .53, *p* = .59), and unemployment (Pearson *χ*^2^(2) = .87, *p* = .65).

**Table 1:** Descriptive statistics of key variables for each personality type.

	Exposure to neighbourhood poverty (*N* = 249)				Work commitment (*N* = 232)				Unemployment (*N* = 249)
	Mean	S.D.	Min.	Max.	Mean	S.D.	Min.	Max.	%
Undercontrollers	.12	.26	0	1	2.66	.71	0	4	10.0
Overcontrollers	.08	.23	0	1	2.66	.74	.60	4	10.8
Resilients	.07	.21	0	1	2.81	.76	.60	4	11.4

Note: The scale anchors for work commitment were 0–4.

To test our hypotheses, we first examined the direct effects of exposure to neighbourhood poverty on work commitment (Table 2: M1) and unemployment (Table 2: M2). We did not find a significant effect of neighbourhood on work commitment. The model for unemployment shows that growing up in poor neighbourhoods was related to greater odds of becoming unemployed in early adulthood. Furthermore, lower educated respondents had greater odds of being unemployed than higher educated respondents. Only one category of parental education was found to be related to work commitment, however, we were not able to find a clear pattern in the parental education variables.

**Table 2:** Models predicting early adulthood work commitment and unemployment.

	M1: Linear regression of work commitment (N = 232)	M2: Logistic regression of unemployment (N = 249)	
	B (SE)	OR	log OR (SE)
Exposure to neighbourhood poverty	-.05 (.03)	1.42	.35 (.17)*
Personality (ref.: resilients)			
Undercontrollers	-.07 (.06)	.99	-.01 (.24)
Overcontrollers	-.09 (.06)	.90	-.11 (.24)
Sex (female)	.03 (.11)	2.06	.72 (.46)
Education	.07 (.05)	.67	-.40 (.17)*
Parental education (ref.: 6) university or higher)			
1) lower vocational education or lower	.22 (.31)	.34	-1.07 (1.20)
2) preparatory middle-level vocational education	.25 (.21)	.43	-.83 (.89)
3) middle-level vocational education	.27 (.15)	.74	-.31 (.66)
4) higher general continued education or preparatory scientific education	.23 (.18)	.19	-1.67 (1.22)
5) higher vocational education	.27 (.12)*	.69	-.37 (.56)
Parental unemployment	.30 (.14)	1.09	.09 (.90)
Parents foreign	-.00 (.17)	2.65	.98 (.74)
Intercept	2.30 (.15)**	.25	-1.38 (.56)*

M1: R2 = .07; F = 1.62. M2: McFadden’s Pseudo-R2 = .09; F = 1.39.

Note: Exposure to neighbourhood poverty and personality were standardised.

Note 2: The table shows the pooled results of 20 imputations.

** p < .01

* p < .05. a.

In Table 3 we included interaction effects between personality types and exposure to neighbourhood poverty in order to examine if there were different neighbourhood effects for adolescents with different personalities. Both the model predicting work commitment (Table 3: M1) and the model predicting unemployment (Table 3: M2) revealed significant interaction effects between personality (i.e., undercontrollers vs. resilients) and neighbourhood poverty. The model predicting work commitment also showed a significant interaction effect between personality (i.e., overcontrollers vs. resilients) and neighbourhood poverty. Education remained consistently significant in the model predicting unemployment, as did parental education in the model predicting work commitment. Furthermore, in the model predicting work commitment, parental unemployment was significant: respondents who experienced parental unemployment during their youth were more likely to have higher work commitment in early adulthood.

**Table 3:** Interaction effects between adolescent personality types and exposure to neighbourhood poverty on early adulthood work commitment and unemployment.

	M1: Linear regression of work commitment (N = 232)	M2: Logistic regression of unemployment (N = 249)	
	B (SE)	OR	log OR (SE)
Exposure to neighbourhood poverty	-.02 (.03)	1.24	.21 (.17)
Personality (ref.: resilients)			
Undercontrollers	-.07 (.06)	.93	-.07 (.27)
Overcontrollers	-.10 (.06)	.86	-.16 (.25)
Neighbourhood poverty*Undercontrollers	-.09 (.03)**	1.50	.40 (.20)*
Neighbourhood poverty*Overcontrollers	-.07 (.03)*	1.38	.32 (.17)
Sex (female)	.01 (.11)	2.37	.86 (.50)
Education	.07 (.05)	.69	-.38 (.18)*
Parental education (ref.: 6) university or higher)			
1) lower vocational education or lower	.22 (.31)	.34	-1.09 (1.20)
2) preparatory middle-level vocational education	.25 (.21)	.49	-.72 (.89)
3) middle-level vocational education	.25 (.15)	.86	-.15 (.65)
4) higher general continued education or preparatory scientific education	.24 (.18)	.18	-1.70 (1.25)
5) higher vocational education	.22 (.12)*	.79	-.23 (.56)
Parental unemployment	.34 (.14)*	.83	-.18 (1.17)
Parents foreign	-.07 (.17)	3.91	1.36 (.75)
Intercept	2.34 (.16))**	.18	-1.71 (.62))**

M1: R2 = .08; F = 3.46**. M2: McFadden’s Pseudo-R2 = .11; F = 1.49.

Note: Exposure to neighbourhood poverty and personality were standardised.

Note 2: The table shows the pooled results of 20 imputations.

** p < .01

* p < .05; a.

The results in Table 3 showed that adolescent personality moderated the associations between neighbourhood poverty and occupational outcomes. After finding interaction effects, we calculated simple slopes of the effects of neighbourhood poverty for the different personality types [48]. Resilient youth were not affected by neighbourhood poverty in their unemployment (b = .21; s.e. = .16; p = n.s.) and their educational commitment (b = -.02; s.e. = .03; p = n.s.). However, there was a significant negative relation between exposure to neighbourhood poverty and work commitment for overcontrollers (b = -.09; s.e. = .04; p < .05) and undercontrollers (b = -.11; s.e. = .03; p < .01). Moreover, undercontrollers who had a longer exposure to neighbourhood poverty in their formative years had higher unemployment in early adulthood (b = .62; = s.e. = .23; p < .01). Furthermore, although we did not find a difference between overcontrollers and resilients in the unemployment model, the simple slope showed that overcontrollers who were exposed longer to neighbourhood poverty had higher unemployment in early adulthood (b = .67; s.e. = .26; p < .05). In sum, the results show that respondents’ personality type moderated the association between exposure to neighbourhood poverty and both work commitment and unemployment.

## Discussion and Conclusion

In this paper, we set out to examine whether exposure to neighbourhood poverty during middle-to-late adolescence (between ages 16–21) predicted work commitment and unemployment in young adults (aged 25). Subsequently, we studied how this neighbourhood effect differed for individuals with different personality types. In the model without moderation by personality, we did not find an association between neighbourhood poverty and work commitment, but we did find an association between neighbourhood poverty and unemployment. However, when examining the moderating role of the three personality types (undercontrollers, overcontrollers, and resilients), we found that adolescents with different personality types are affected differently by their neighbourhood.

The finding that undercontrollers’ and overcontrollers’ work commitment was weaker and undercontrollers’ odds of unemployment were higher with longer exposure to neighbourhood poverty during adolescence is in accordance with our hypotheses. That is, individuals with a non-resilient personality are more vulnerable to negative environments. These results are consistent with the findings of previous studies showing that individuals who score low on resiliency are particularly vulnerable to contextual factors such as negative parenting behaviour, negative peer environments, and low friendship quality [40,49–51]. These findings underscore the need for research that would clarify why non-resilient individuals are more vulnerable to negative environments than resilients. It may be that resilients, who are in general adaptive, flexible, and intelligent [52], are better able to understand the dominant norm in society that work is important, and are therefore better able to resist the influence of negative norms and attitudes in the neighbourhood. Overcontrollers and undercontrollers, however, have difficulties in adapting to societal norms, and may therefore more easily fall back on norms that are dominant in a more proximate area. Thus, they may be more likely to absorb the negative norms and attitudes about work when growing up in poor neighbourhoods, leading to low work commitment or unemployment.

We hypothesised that only the non-resilient personality types (undercontrollers and overcontrollers) would be affected by exposure to neighbourhood poverty. We found this for both undercontrollers and overcontrollers in the model for work commitment and for undercontrollers in the model for unemployment. However, we could not find a difference between overcontrollers and resilients in their association between exposure to neighbourhood poverty and unemployment risk. Despite the non-significant interaction effect, when comparing the interaction coefficients of overcontrollers and undercontrollers, the pattern looks highly similar. Furthermore, when we calculated the simple slope for overcontrollers, we found a significant relation between neighbourhood poverty and unemployment. The results suggest that the differences were not between undercontrollers and overcontrollers, but rather between resilients and non-resilients. The reason we did not find the significant interaction for overcontrollers could be because of the limited sample size.

A frequent problem in neighbourhood effects studies is selection bias; that is, individual characteristics that influence neighbourhood choice may also influence the studied outcome variables. When individual characteristics are not controlled for in the model, neighbourhood effects may therefore reflect the effect of the individual characteristics. In our analyses we had the advantage to study adolescent respondents. Given that it is generally the parents and not the adolescents who decide where to move, selection effects of the adolescents’ individual characteristics are unlikely. However, this does not preclude an intergenerational selection effect through the parents (see also [53]). For example, unemployed parents may have certain economic constraints, forcing them to choose a relatively disadvantaged neighbourhood with lower house prices. This same parental unemployment may also affect the child because they do not have positive role models in the home environment, possibly leading to negative work attitudes and even unemployment later in life. We attempted to control for this by including parental education, parental unemployment, and parental ethnicity in the models, which give some information about the home environment. Unfortunately, a more direct measure such as parental income was not available. Furthermore, longitudinal measurements of our dependent variables were not available, excluding modeling possibilities such as fixed-effects models, which can better deal with selection issues. Therefore, it is possible that an intergenerational selection effect may still bias the results to some extent. Despite that, our analyses suggest different vulnerabilities to neighbourhood effects for individuals with different personality types. Future research would benefit from longitudinal analyses to further deepen our understanding of individual responses to neighbourhood adversity.

In sum, work commitment and unemployment of resilient individuals are not predicted by neighbourhood poverty, whereas the work commitment and unemployment of individuals with non-resilient personality types (i.e., overcontrollers and undercontrollers) are. Our findings underline the importance of studying different vulnerabilities of personality types in distal environments such as the neighbourhood, compared to more commonly studied proximate environments such as the family. Furthermore, it calls into question the efficiency of neighbourhood based intervention policies, where a more specific focus on at-risk individuals may be more appropriate.

## Supporting Information

S1 File This is the supporting data file. (DTA)

## References

[1] Dietz RD. The estimation of neighborhood effects in the social sciences: An interdisciplinary approach. Soc Sci Res. 2002;31: 539–575.

[2] Durlauf SN. Neighbourhood effects In: Henderson JV, Thisse JF, editors. Handbook of regional and urban economics Volume 4, Cities and Geography. Amsterdam: Elsevier; 2004.

[3] Ellen IG, Turner MA. Does neighbourhood matter? Assessing recent evidence. Hous Policy Debate. 1997;8(4): 833–866.

[4] Galster G. An economic efficiency analysis of deconcentrating poverty populations. J Hous Econ. 2002;11(4): 303–329.

[5] van Ham M, Manley D, Bailey N, Simpson L, Maclennan D. New perspectives In: van Ham M, Manley D, Bailey N, Simpson L, Maclennan D, editors. Neighbourhood effects research: New perspectives. Dordrecht: Springer; 2012 pp. 1–22.

[6] Brattbakk I, Wessel T. Long-term neighbourhood effects on education, income and employment among adolescents in Oslo. Urban Stud. 2013;50(2): 391–406.

[7] Manley D, van Ham M. Neighbourhood effects, housing tenure and individual employment outcomes In: van Ham M, Manley D, Bailey N, Simpson L, Maclennan D, editors. Neighbourhood effects research: New perspectives. Dordrecht: Springer; 2012 pp. 147–174.

[8] Galster GC. The mechanism(s) of neighbourhood effects: Theory, evidence, and policy implications In: van Ham M, Manley D, Bailey N, Simpson L, Maclennan D, editors. Neighbourhood effects research: New perspectives. Dordrecht: Springer; 2012 pp. 23–56.

[9] Akers RL, Krohn MD, Lanza-Kaduce L, Radosevich M. Social learning and deviant behavior: a specific test of a general theory. Am Sociol Rev. 1979;44(4): 636–655. 389120

[10] Friedrichs J, Blasius J. Social norms in distressed neighbourhoods: Testing the Wilson hypothesis In: Friedrichs J, Galster G and Musterd S (eds) Life in poverty neighbourhoods. London & New York: Routledge; 2005 pp. 11–30.

[11] Wilson WJ. The truly disadvantaged Chicago: Chicago University Press; 1987.

[12] van de Wouden R, de Bruijne E. De stad in de omtrek [The city in the perimeter] The Hague: The Netherlands Institute for Social Research; 2001.

[13] Kifle T. Relative income and job satisfaction: Evidence from Australia. Appl Res Qual Life. 2013;8: 125–143.

[14] Larson RW, Richards MH, Moneta G, Holmbeck G, Duckett E. Changes in adolescents’ daily interactions with their families from ages 10 to 18: Disengagement and transformation. Dev Psychol. 1996;32: 744–754.

[15] Kearns A, Parkinson M. The significance of neighbourhood. Urban Stud. 2001;38(12): 2103–2110.

[16] Buck N. Identifying neighborhood effects on social exclusion. Urban Stud. 2001;38(12): 2251–2275.

[17] Galster G, Marcotte DE, Mandell M, Wolman H, Augustine N. The influence of neighborhood poverty during childhood on fertility, education, and earnings outcomes. Hous Stud. 2007;22(5): 723–751.

[18] Musterd S, Galster G, Andersson R. Temporal dimensions and measurement of neighbourhood effects. Environ Plan A. 2012;44: 605–627.

[19] Meeus W, Dekovic M, Iedema J. Unemployment and identity in adolescence: A social comparison perspective. Career Dev Q. 1997;45: 369–380.

[20] Branje S, Laninga-Wijnen L, Yu R, Meeus W. Associations among school and friendship identity in adolescence and romantic relationships and work in emerging adulthood. Emerg Adulthood. 2014;2(1): 6–16.

[21] Yu R, Nieuwenhuis J, Meeus W, Hooimeijer P, Koot HM, Branje S. Biological sensitivity to context: Cortisol awakening response moderates the effects of neighbourhood density on the development of adolescent externalizing problem behaviours. Biol Psychol. 2016;120: 96–107. 10.1016/j.biopsycho.2016.08.004 27543043 PMC5074006

[22] Block JH, Block J. The role of ego-control and ego-resiliency in the organization of behavior In: Collins WA, editors. Development of cognition, affect, and social relations (Vol. 13). Hillsdale: Lawrence Erlbaum Associates; 1980.

[23] Nieuwenhuis J, Hooimeijer P, Meeus W. Neighbourhood effects on educational attainment of adolescents, buffered by personality and educational commitment. Soc Sci Res. 2015;50: 100–109. 10.1016/j.ssresearch.2014.11.011 25592923

[24] Nieuwenhuis J, Hooimeijer P, van Ham M, Meeus W. Neighbourhood effects on migrant youth’s educational commitments, an enquiry into personality differences. Urban Stud. In press. 10.1177/0042098016640693 PMC5519151 28781388

[25] Netherlands Statistics. Statline: Bevolking naar herkomst en generatie [Statline: Population according to origin and generation] Voorburg: Statistics Netherlands; 2015.

[26] Caspi A, Moffitt TE, Thornton A, Freedman D, Amell JW, Harrington H, et al The life history calendar: A research and clinical assessment method for collecting retrospective event-history data. Int J Methods Psychiatr Res. 1996;6, 101–114.

[27] Crocetti E, Rubini M, Meeus WHJ. Capturing the dynamics of identity formation in various ethnic groups: Development and validation of a three-dimensional model. J Adolesc. 2008;31: 207–222. 10.1016/j.adolescence.2007.09.002 17959238

[28] CBS. Kerncijfers postcodegebieden, 2004 [Key figures for postcode areas, 2004] The Hague: Statistics Netherlands; 2006.

[29] Visser P, van Dam F, Hooimeijer P. Residential environment and spatial variation in house prices in the Netherlands. Tijdschr Econ Soc Geogr. 2008;99(3): 348–360.

[30] Oberwittler D, Wikström POH. Why small is better: Advancing the study of the role of behavioral contexts in crime causation In: Weisburd WB, Bruinsma G, editors. Putting crime in its place. New York, NY: Springer; 2009 pp. 33–58.

[31] Goldberg LR. The development of markers for the Big-Five factor structure. Psychol Assess. 1992;4: 26–42.

[32] Vermulst AA, Gerris JRM. QBF: Quick Big Five persoonlijkheidstest handleiding [Quick Big Five personality test manual] Leeuwarden, the Netherlands: LDC Publications; 2005.

[33] Branje SJ, van Lieshout CF, Gerris JR. Big Five personality development in adolescence and adulthood. Eur J Pers. 2007;21: 45–62.

[34] Robins RW, John OP, Caspi A, Moffitt TE, Stouthamer-Loeber M. Resilient, overcontrolled, and undercontrolled boys: Three replicable personality types. J Pers Soc Psychol. 1996;70(1): 157–171. 8558407 10.1037//0022-3514.70.1.157

[35] Klimstra TA, Hale WW III, Raaijmakers QAW, Branje SJT, Meeus WHJ. A developmental typology of adolescent personality. Eur J Pers. 2010;24: 309–323.

[36] Meeus W, van de Schoot R, Klimstra T, Branje S. Personality types in adolescence: Change and stability and links with adjustment and relationships: A five-wave longitudinal study. Dev Psychol. 2011;47: 1181–1195. 10.1037/a0023816 21639626

[37] Nagin DS. Group-based modeling of development Cambridge, MA: Harvard University Press; 2005.

[38] Branje SJ, Hale WW III, Frijns T, Meeus WH. Longitudinal associations between perceived parent-child relationship quality and depressive symptoms in adolescence. J Abnorm Child Psychol. 2010;38: 751–763. 10.1007/s10802-010-9401-6 20217211 PMC2902740

[39] Asendorpf JB, van Aken MA. Personality–relationship transaction in adolescence: Core versus surface personality characteristics. J Pers. 2003;71: 629–666. 12901433 10.1111/1467-6494.7104005

[40] Dubas JS, Gerris JR, Janssens JM, Vermulst AA. Personality types of adolescents: concurrent correlates, antecedents, and type X parenting interactions. J Adolesc. 2002;25: 79–92. 10.1006/jado.2001.0450 12009751

[41] StataCorp. Stata Statistical Software: Release 13. College Station, TX: StataCorp LP; 2013.

[42] Li C. Little’s test of missing completely at random. Stata J. 2013;13(4): 795–809.

[43] Asendorpf JB, van der Schoot R, Denissen JJA, Hutteman R. Reducing bias due to systematic attrition in longitudinal studies: The benefits of multiple imputation. Int J Behav Dev. 2014;38(5): 453–460.

[44] van Buuren S. Multiple imputation of discrete and continuous data by fully conditional specification. Stat Methods Med Res. 2007;16: 219–242. 10.1177/0962280206074463 17621469

[45] Rubin DB. Multiple imputation for nonresponse in surveys. New York: Wiley; 1987.

[46] Nieuwenhuis J, Hooimeijer P. The association between neighbourhoods and educational achievement, a systematic review and meta-analysis. J Hous Built Environ. 2016;31(2): 321–347. 29355196 10.1007/s10901-015-9460-7 PMC5748572

[47] Nieuwenhuis J. Publication bias in the neighbourhood effects literature. Geoforum. 2016;70: 89–92.

[48] Aiken LS, West SG. Multiple regression: Testing and interpreting interactions Newbury Park, CA: Sage; 1991.

[49] Yu R, Branje S, Keijsers L, Koot HM, Meeus W. Pals, problems, and personality: the moderating role of personality in the longitudinal association between adolescents’ and best friends’ delinquency. J Pers. 2013;81(5): 499–509. 10.1111/jopy.12027 23301765

[50] O'Connor BP, Dvorak T. Conditional associations between parental behavior and adolescent problems: A search for personality–environment interactions. J Res Pers. 2001;35: 1–26.

[51] van Aken MA, Semon Dubas J. Personality type, social relationships, and problem behaviour in adolescence. Eur J Dev Psychol. 2004;1: 331–348.

[52] Block J, Kremen AM. IQ and ego-resiliency: conceptual and empirical connections and separateness. J Pers Soc Psychol. 1996;70(2): 349–361. 8636887 10.1037//0022-3514.70.2.349

[53] van Ham M, Hedman L, Manley D, Coulter R, Östh J. Intergenerational transmission of neighbourhood poverty. An analysis of neighbourhood histories of individuals. Trans Inst Br Geogr. 2014;39: 402–417. 10.1111/tran.12040 26074624 PMC4459034

